# Controllable Chiral Light Generation and Vortex Field Investigation Using Plasmonic Holes Revealed by Cathodoluminescence

**DOI:** 10.1021/acs.nanolett.3c04262

**Published:** 2024-01-04

**Authors:** Takumi Sannomiya, Taeko Matsukata, Naoki Yamamoto

**Affiliations:** † Department of Materials Science and Technology, Tokyo Institute of Technology, 4259 Nagatsuta Midoriku, Yokohama 226-8503, Japan

**Keywords:** nanohole, surface plasmons, cathodoluminescence, scanning transmission electron microscopy, circularly polarized light, transition radiation

## Abstract

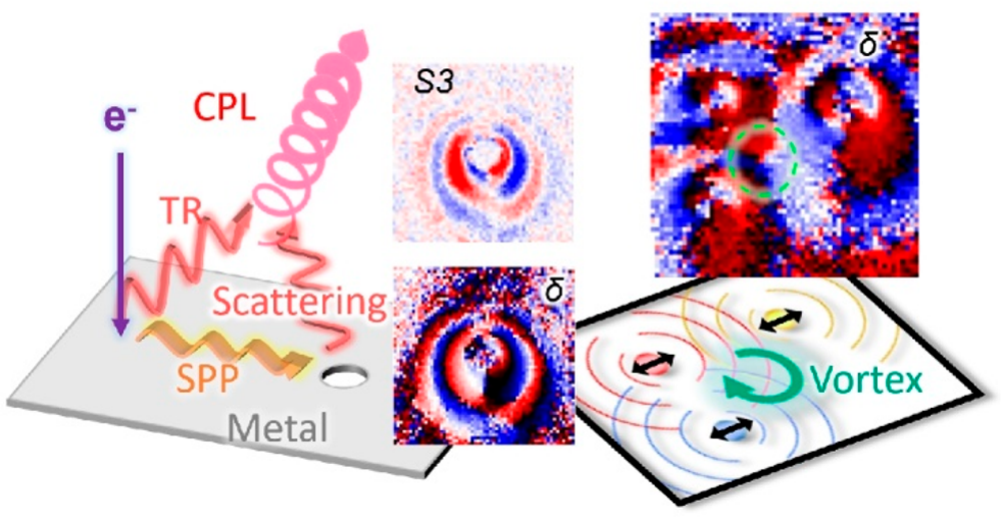

Control of the angular momentum of light is a key technology for next-generation nano-optical devices and optical communications, including quantum communication and encoding. We propose an approach to controllably generate circularly polarized light from a circular hole in a metal film using an electron beam by coherently exciting transition radiation and light scattering from the hole through surface plasmon polaritons. The circularly polarized light generation is confirmed by fully polarimetric four-dimensional cathodoluminescence mapping, where angle-resolved spectra are simultaneously obtained. The obtained intensity and Stokes maps show clear interference fringes as well as almost fully circularly polarized light generation with controllable parities by the electron beam position. By applying this approach to a three-hole system, a vortex field with a phase singularity is visualized in the middle of three holes.

Control of angular momentum of light is one of the crucial technologies to realize next-generation nano-optical devices and optical communications, including quantum communication and encoding,^1−4^ and has been extensively investigated in topological photonic structures or for coupling to the electron spins in materials. Spin angular momentum (SAM) of light is represented by left- or right-handed circularly polarized light (CPL), which is a robust signal carrier and has already been practically utilized, e.g., in 3D cinemas to send two distinct signals for right and left eyes regardless of the observation angle. For a macroscopic CPL parity control, a linear polarizer and a quarter waveplate are typically combined as a set of filters, which require much larger scale elements than the light wavelength. For micro- or nanoscale devices, it has been proposed to couple a chiral optical nanoantenna with a light source to select the CPL parity by the structure.^5−7^ This antenna coupling strategy is useful because a linearly polarized light source can be utilized instead of a CPL-selective source, which is not readily available, when coupled with such chiral antennas. However, when the light source is fixed to the structure, dynamic control of the CPL is not straightforward because the chirality is fixed to the antenna geometry. To overcome this problem, achiral nanoantennas combined with an optical waveguide have been proposed, where the optical signal from different directions along the waveguide determines the generated CPL parity.^8^ This is based on an elaborate fabrication of optical waveguides with coupled nanoantennas, and the highly efficient propagation of light is required. As an alternative approach, CPL generation using an electron beam, or cathodoluminescence (CL), has been proposed, which is possible even from a spherical particle by controlling the phase difference of two dipoles in the structure.^9−11^ Such electron beam-based light generation is expected to be a scheme for prospective laser or quantum light sources.^12,13^ Although the use of the electron beam for optical devices requires different technologies compared to purely light-based systems and may give limitations, the electron-beam approach is not unrealistic as there have been various table-top devices with electron beams, such as cathode-ray tubes, streak-cameras, and short-wavelength light sources, indicating the practical applicability of electron beams. While integrated photonics facilitate the production of the light sources and would realize photonic circuits,^7,8,14^ electron-beam-based technology can offer extremely fast switching,^12^ as is already used in fast cameras.

Here we propose a new approach to generate CPL using circular metallic holes coupled to surface plasmon polaritons (SPPs), which is a different mechanism compared to the previous studies. Plasmonic hole structures are useful antennas since SPPs are already incorporated in the structure, working as a guided wave on the surface. In such structures, transition radiation (TR) and a SPP scattering field, which are generated at spatially separate locations, lead to interference; When an electron beam is irradiated onto the metal film, away from the antenna structures, TR is generated at the electron beam position and simultaneously and coherently excited SPPs are scattered by the antenna and create photons. Since all the interaction processes are coherent with respect to the electron excitation, the light from these two sources interferes.^15^ Such interferences with TR have given a way to access the phase of the field in the CL measurement.^16^ In this study, we utilized this interference of TR and SPP scattering light to control CPL (Figure 1a). Also using this CPL generation tool with an electron beam, we further visualize a vortex field generation from three holes, emulating a three-phase optical field motor, which corresponds to the orbital angular momentum (OAM) in the SPP field.

**Figure 1:**
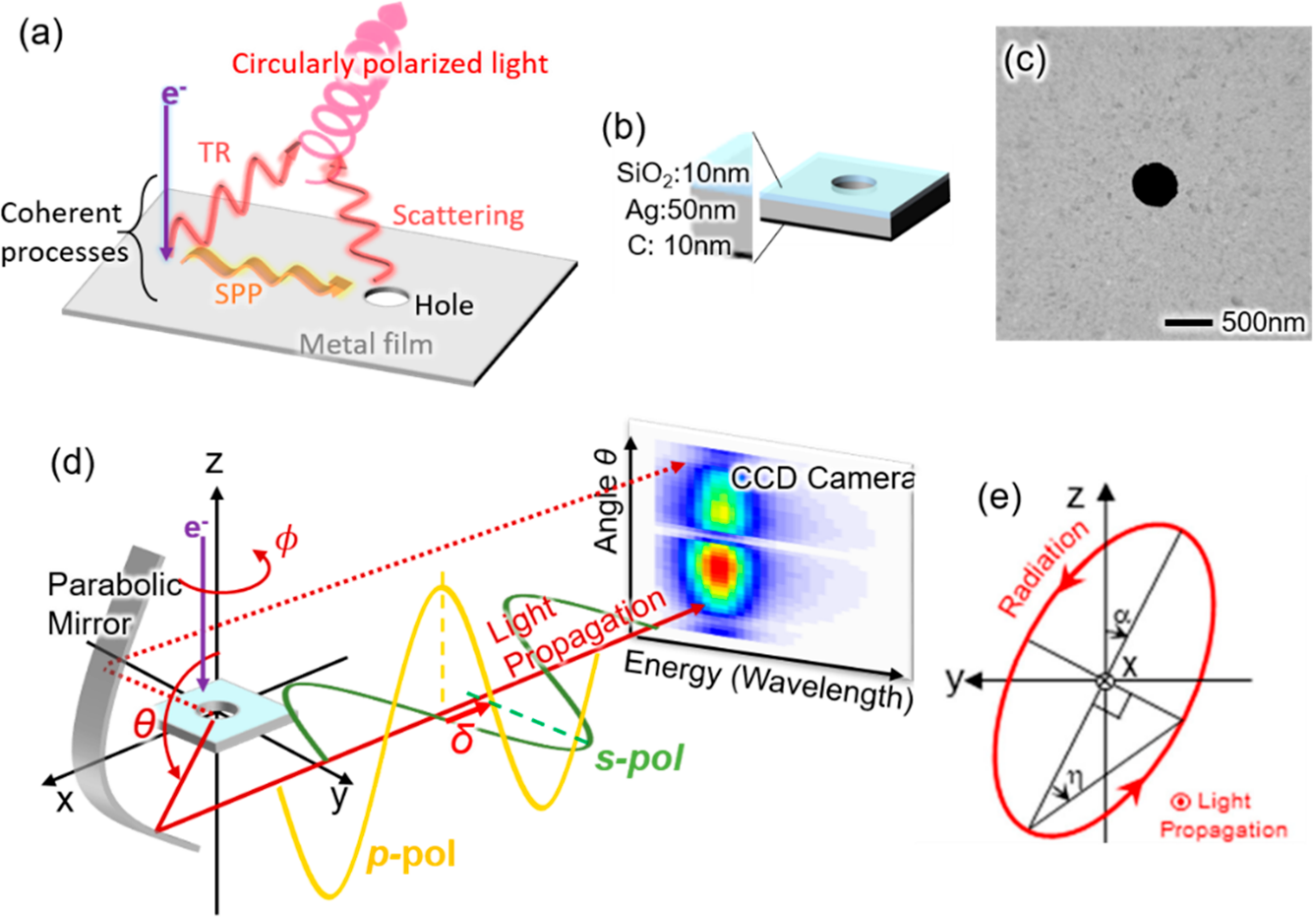
Concept and method of the study. (a) Illustration of circularly polarized light generation through interference of transition radiation (TR) and surface plasmon polariton (SPP) scattering from a hole excited by an electron beam. (b) Dimension of the free-standing nanohole membrane used in the study. (c) STEM dark field image of the fabricated 500 nm hole. (d) Illustration of the fully polarimetric four-dimensional cathodoluminescence measurement. The coordinate, angle, and polarization definitions are also shown. (e) Definition of polarization parameters.

Holes are fabricated on a free-standing silver membrane by colloidal lithography with a film transfer method.^17,18^ The “upside” of the silver membrane is covered by SiO_2_ to avoid degradation, and the “downside” is covered by carbon so that SPP propagates only along the upside of the membrane (Figure 1b). A scanning transmission electron microscopy (STEM) CL system is used to evaluate the CPL generation from circular silver holes. The four-dimensional (4D) Stokes data acquisition in this setup allows simultaneous angle- and wavelength-resolved CL mapping, as illustrated in Figure 1d,e.^9^

Figure 2 shows CL mapping results of a 500 nm silver hole with *p*-polarization at various detection angles θ and photon wavelengths. These CL maps are obtained by scanning the electron beam two-dimensionally on the sample, and the CL signal is plotted in synchronization with the beam scan. The left and right halves of the CL maps with symmetric detection angles are shown, namely pairs of θ = 45°, 135° and θ = 30°, 150°, facing each other in order to compare their interference fringe patterns. The interference fringe pattern is formed clearly at the top and bottom of the images with different fringe pitches, which are also dependent on the wavelength and detection angles. These fringe patterns can be understood as the interference between TR and the scattering from the hole through SPP propagation, as schematically illustrated in Figure 2a.^16^ The phase difference φ of TR and the scattered wave from the hole generate the interference fringe patterns. The phase difference φ can be expressed using the electron beam position *R* from the center of the hole as
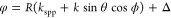
1where *k*_spp_ is the SPP wave vector, *k* is the free-space light wave vector, and ϕ is the azimuthal angle of the position. Δ corresponds to an additional phase shift related to the excitation of TR and the scattering of SPP by the hole. The interference patterns have shorter pitches in the upper half of the image (*x* > 0) than the lower half (*x* < 0), which is because the sign of the TR phase shift (second term in eq 1) flips with respect to the light through SPP scattering at the hole (first term in eq 1), as illustrated in Figure 2a. The comparison of the maps with symmetric detection angles from the sample plane reveals that the fringe patterns do not match for the symmetric pairs (θ = 45°, 135° pair in Figure 2b and θ = 30°, 150° pair in Figure 2c). This mismatch is related to the phase difference of the TR toward the top (θ < 90°) and bottom (θ > 90°) spaces, contributing to the offset Δ in eq 1. The phase difference between the top-ward and the bottom-ward TRs is approximately π when the space is separated by an ideal metal film producing mirror-symmetric dipole radiations.

To better understand the behavior of TR, we calculated the electromagnetic field distribution by 80 keV electron moving in and out of a silver surface, as shown in Figure 2d.^19^ TR is emitted into the free space and SPPs propagate on the surface, as described in the scheme of Figure 2a. Since the TR is an effective electric dipole polarized perpendicular to the interface, the transverse magnetic field, as plotted in Figure 2d, represents well the phase of the TR field. The zero phase is set when the electron is located at the interface. As clearly seen, the phase difference is ∼π, which can be slightly shifted due to the presence of the carbon layer. The theoretically calculated TR phase from a carbon surface is also shown in the Supporting Information. While TR can be approximated by a perpendicular electric dipole, the field from a hole in a metallic film can be described by a magnetic dipole,^20−23^ which, e.g., has the magnetic polarization along the *y*-axis when the electron beam is placed on the *x*-axis as in the configuration of Figure 2a. This scattering field from the hole magnetic dipole gives symmetric magnetic distributions on the top (*z*-positive) and back (*z*-negative) sides of the sample, as shown on the right of Figure 2d. Thus, the radiation by TR and hole scattering differently interferes in the top (*z*-positive) and back (*z*-negative) side hemispheres of the space, generating shifted interference patterns as in the experiment in Figure 2b,c. In the upside-down configuration of the sample, as shown in Figure 2e, the interference pattern shifts only slightly. This indicates that the phase shifts of the SPP and TR excitation by flipping the sample cancel out the total phase offset Δ together with the influence of the carbon layer.

Considering that the interference between TR and SPP-mediated scattering gives nice fringe patterns for *p*-polarization mapping revealing the phase difference between TR and hole scattering, one can utilize this interference to produce CPL when the *s*-polarization component in the hole scattering is included. Although CPL cannot be produced in a system with a point symmetry, the symmetry of this circular nanohole system can be broken by the electron beam position, detection angle, and polarization, i.e., by so-called extrinsic chirality.^9,10^ We analyze these CPL properties by calculating Stokes parameters from six CL mapping images with different polarizations, namely, *p*-polarization, *s*-polarization, +45°-polarization, −45°-polarization, RCP, and LCP (see Figure 1d,e for the parameter definition). The six images with different polarizations are obtained separately by scanning the electron beam over the same area of the sample and the exact position of each image is aligned and normalized for the Stokes parameter calculation. More details of the procedure is described in the Supporting Information as well as in the previous study.^9^ The representative Stokes mapping results are shown in Figure 3 for different detection angles and wavelengths. In all of the maps, interference fringe patterns due to TR and hole scattering are visible. Similarly to the interference patterns in Figure 2, these fringe patterns become asymmetric with respect to the *y*-axis (horizontal axis) when the detection angle is off the *z*-axis, as suggested by eq 1.

**Figure 2:**
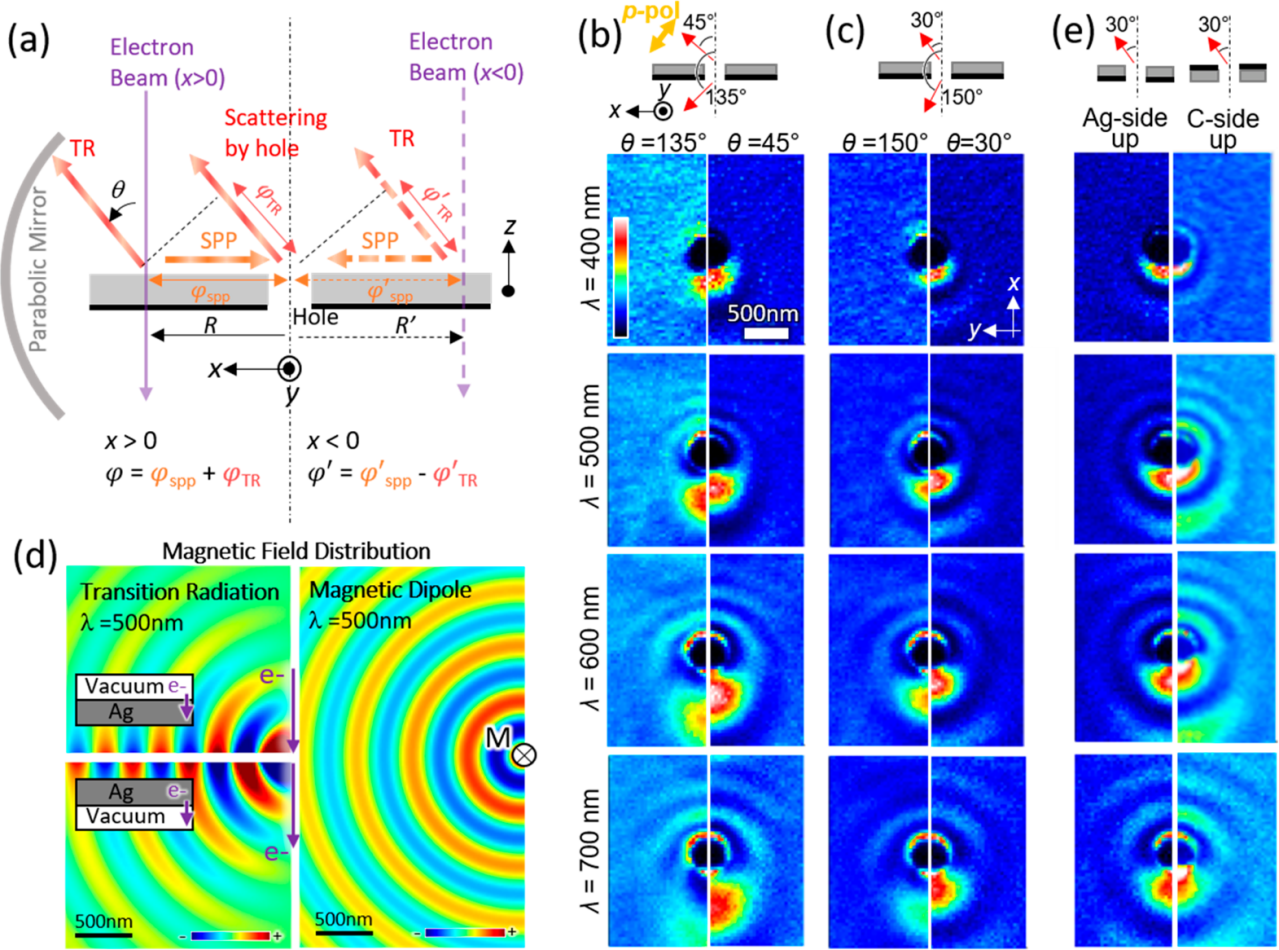
CL mapping of a 500 nm single silver hole with *p*-polarization, i.e., polarization in the *x*-*z* plane. (a) Schematic illustration of the interference between TR and scattering by the hole through SPPs projected on the *x*-*z* plane. The condition, where the electron beam is incident at negative *x* positions, is indicated by dashed arrows and primes of the parameters. (b,c) Mapping results with halves of the images of symmetric detection pairs (b: θ = 45° and 135°, c: θ = 30° and 150°) facing each other so that the fringe patterns can be directly compared. (d) Calculated magnetic field perpendicular to the screen for TR (left) and for a magnetic dipole polarized perpendicular to the screen (right) for the vacuum wavelength of 500 nm. TR is plotted for the top (*z*-positive) and back (*z*-negative) sides of a bulk silver surface by 80 keV electron impact. (e) θ = 30° measurement maps with the silver (left) and carbon (right) sides up. The mappings (b,c,e) are obtained by scanning the focused electron beam on the *x*-*y* plane on the sample, and the image intensities are integrated over ±25 nm in the wavelength and ±2.5° in the angle θ.

In the S3 (RCP-LCP signal) maps in Figure 3, both LCP (blue) and RCP (red) generations are confirmed, which are all mirror-symmetric with a flipped sign with respect to the *x-z* plane (vertical line at the center of the image). This symmetry can be understood from the inclusion of the mirror-symmetric electric field with a flipped sign against the *x-z* plane of the hole magnetic dipole polarized along the *x* direction when the electron beam is located off the center of the hole. Since the electric field of TR is symmetric with respect to the *x-z* plane, the interference between the TR and hole scattering generates CPL together with the interference fringe patterns corresponding to the phase difference between TR and hole scattering.

**Figure 3:**
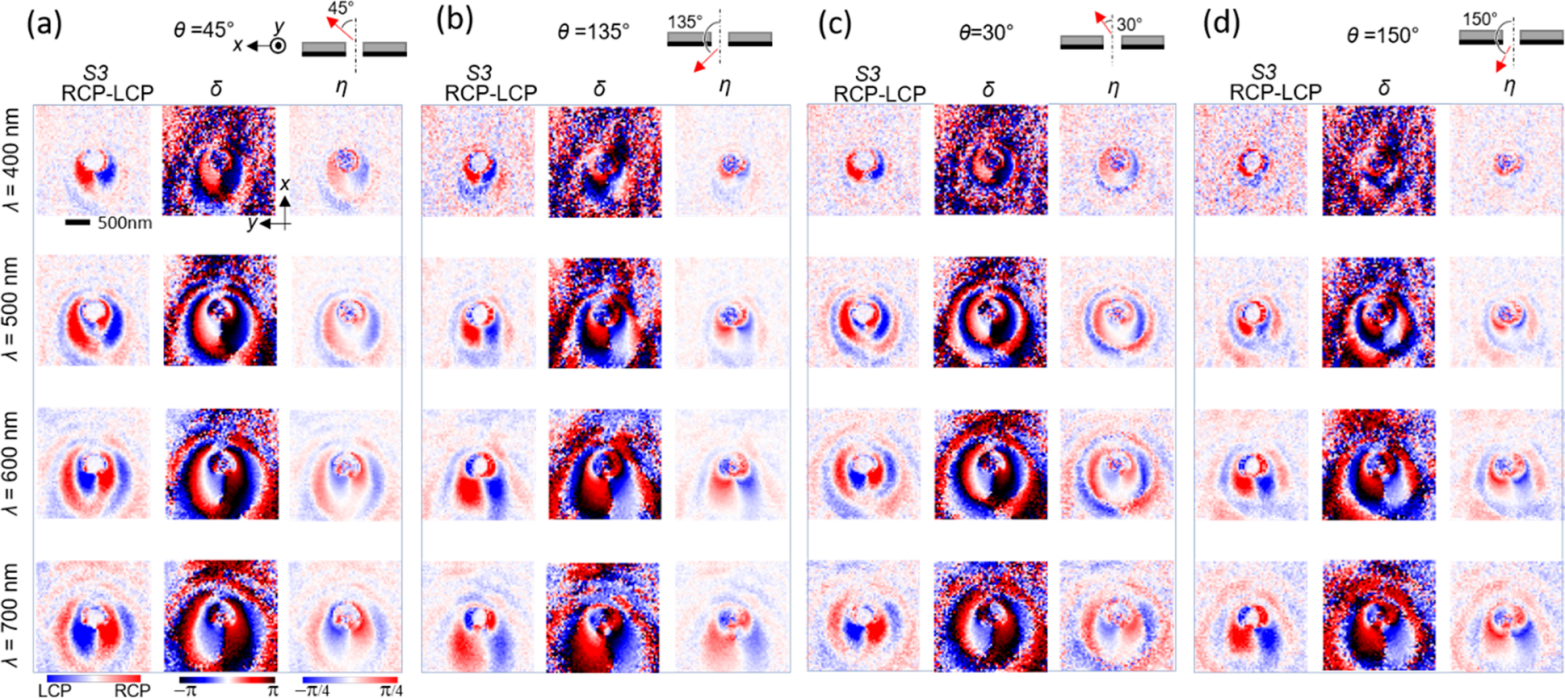
Circularly polarized light generation from a 500 nm silver hole under various conditions. Six CL maps with different polarizations are obtained by scanning the focused electron beam on the *x*-*y* plane of the sample, and Stokes parameters are calculated at each pixel after adjusting the images positions. Stokes mappings of *S3* (difference of RCP and LCP map intensities), δ, and η are performed for the detection angles of (a) θ = 45°, (b) θ = 135°, (c) θ = 30°, and (d) θ = 150°. Maps of representative photon wavelengths are given in the rows.

The δ maps, showing the phase difference between the *p*- and *s*-polarization components, and the η maps, corresponding to CPL “circularity”, show that almost perfect CPL (δ = ±π/2 with η = ±π/4) is available (see Figure 1d,e for the parameter definition). Under certain conditions especially with the electron beam situated at the bottom half of the image or near the hole, fully circular CPL can be observed. This shows that the polarization parameters of the generated CPL can be controlled by the electron beam position for certain detection angles. Thanks to the SPP decay in the 2D planner space, the CPL intensity can also be controlled by the electron beam position by locating the beam closer or further to the hole (see Supporting Information for the intensity discussion).

While the radiation field from a hole in a metal film corresponds to the field distribution of a magnetic dipole,^20−23^ the SPP field on the film generated by the hole can be treated as an in-plane-polarized electric dipole in the 2D space. We now utilize the hole dipoles on the surface as SPP field sources to generate a vortex field on a surface, corresponding to the OAM in the SPP field, and visualize this field rotation by Stokes mapping. We demonstrate this vortex generation using three holes, as shown in Figure 4. We chose this configuration with one large hole and two smaller ones to ascertain a strong SPP scattering field in the middle of the three with different phases to create a vortex. When a plane wave is illuminated, such holes within the illumination field will be coherently excited with some phase differences for a given illumination angle. Due to the reciprocity of the wave, CL measurement with a certain detection angle *θ* corresponds to the *z*-field measurement generated by such plane wave illumination with an angle *θ,* as illustrated in Figure 4a. (see also the Supporting Information) Thus, we can reasonably analyze the wave interference from the three holes by CL field mapping as the generated field from the holes in the reciprocal manner. Such a rotating field from three field sources with certain phase differences can be understood from the concept of a three-phase motor, as described in Figure 4b.

The measured CL maps with θ = 30° and λ = 660 nm are shown in Figure 4c–g for different polarization conditions and representative Stokes parameters. The Stokes maps are reproduced from six images of different polarizations by scanning the electron beam over the sample in the same manner as in the results of Figure 3. Clearly, interferences of SPPs from the holes are observed in the positions between the holes, generating a more complicated field distribution compared to the single hole case. Closely looking at the *s*-polarization field map and the δ map (Figure 4g), one finds a position with a phase singularity in the middle of the three holes, which is marked by dashed circles. At this position, we expect field interference from these three sources. The phase pattern (Figure 4g) gives a rotating phase, and no amplitude (Figure 4d) at the center of this rotation is found, which are the typical features of the phase singularity of the vortex fields.

To compare the experimental results and confirm the vortex field, analytically calculated field distributions are shown in Figure 4h–m. In this model, three dipole sources on a 2D plane are excited by a plane wave with an incidence angle of θ = 30°, which is reciprocal to the CL measurement (Figure 4a). To introduce the additional field of the TR in the analytical calculation, a plane wave field over the surface of the calculation plane is overlaid (see calculation details in the Supporting Information). The analytical calculation patterns in Figure 4j-m well reproduce the experimental results, as seen in the pitch and position of the interference fringes in the *p*-polarization and S3 maps (Figure 4d,e,j,k) as well as the field singularity in the *s*-polarization maps (Figure 4d,j) at the vortex position indicated by the circle and the vortex phase rotation in the δ phase maps (Figure 4g,m). Although only the phase difference of *s*- and *p*-polarizations (not each phase) is available in the experiment, according to the analytical calculation, the *p*-polarization phase in Figure 4i is dominated by TR (plane wave on the surface) constantly shifting in the *x* direction, with almost no local variation originating from the *p*-polarized dipole field from each hole. Thus, this CPL phase measurement basically extracts the phase of the *s*-polarized (horizontally polarized) dipoles. The local variation of the relative phase δ (Figure 4g,m) corresponds to the *s*-polarization phase (Figure 4h) spread on the homogeneous background with a constant inclination of the *p*-polarized TR phase. (Figure 4i).

At the vortex field position, as marked by circles in Figure 4j,m in the middle of the three holes, the simulated singularity features correspond well to the experimental ones in the experiment (Figure 4d,g). Since this field singularity is mainly of the *s*-polarized (horizontally polarized) dipole fields (Figure 4h), it is natural that only the *s*-polarization field gives zero amplitude at this singularity point (Figure 4d,j) while the field is nonzero at this position in the *p*-polarization field patterns (Figure 4e,k). This singularity at the vortex center is also confirmed by the well-corresponding phase plots of *s*-polarization and δ in Figure 4h,m. In SI, the rotating phase reproduced by the simulation is shown. Thus, we have visualized the SPP vortex field generated from three holes, similar to a three-phase field motor. This demonstrates that such a vortex field can be generated in a much simpler way than highly organized nanostructures.^24^

**Figure 4:**
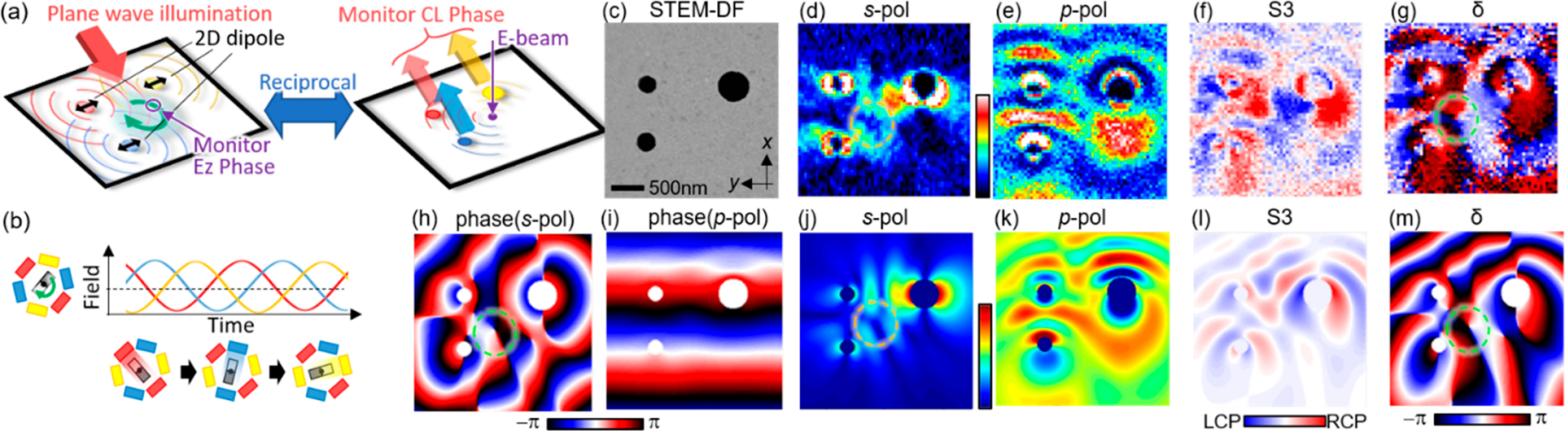
Vortex field generation using three holes. (a) Schematic illustration of monitoring the SPP field rotation generated by three holes with plane wave excitation and corresponding CL detection, which are reciprocal to each other. (b) Illustration of the field rotation from three sources, mimicking a three-phase motor. (c-g) Experimental Stokes maps obtained at the detection angle θ = 30° with the wavelength of 660 nm by scanning the electron beam on the *x*-*y* plane. (h-m) Calculated field patterns with three in-plane dipoles and a propagating plane wave along the *x*-axis in a two-dimensional system, respectively emulating the three holes and the TR in the experiment. The vortex position is indicated by dashed circles in the *s*-polarization (d,j) and phase maps (g,h,m), corresponding to the *s*-polarization field rotation. The vortex center corresponds to a field singularity and has no field amplitude in the *s*-polarization map (d,j).

In conclusion, we successfully demonstrated CPL generation from a hole in a metal film using an electron beam. This CPL is tunable by controlling the interference of TR at the electron beam position and coherent scattering field at the hole through the SPP. The relative phase difference of the TR and hole scattering field determines the CPL parity. The CPL generation is confirmed by the 4D Stokes mapping CL approach by visualizing the Stokes parameter distributions as functions of electron beam position, radiation angle, and wavelength. Using this CPL generation and mapping technique, a SPP vortex field is also demonstrated, where three dipole fields interfere and generate a rotating field, showing phase singularity. The CPL generation mechanism and field control strategy using coherent light generation processes are useful in realizing on-demand CPL sources. Compared to the CPL generation by controlling the electron beam within nanoantennas of subwavelength scales,^9^ the proposed approach requires less precise control of the electron beam size and position, i.e., in the scale of the light wavelength or even larger.

## Methods

For the CL measurement, a modified STEM (2100F, JEOL Japan) instrument equipped with a Schottky type field emission gun and an aberration corrector is operated at 80 kV acceleration with a beam current of 1 nA.^9,25^ The parabolic mirror situated at the sample position collimates the light emission from the sample. Only the ϕ = 0° emission angle component of the emission is selected by the slit mask, while θ = 0–180° component is dispersed on the CCD camera to obtain 2D information on θ and the wavelength at each electron beam position (Figure 1). With the electron beam scan, 4D data sets are obtained. A polarizer and a phase plate are inserted between them for the polarimetric analysis (Supporting Information). The dispersion of the phase plate and the phase shift due to reflection by the mirror are corrected.

## Supporting Information Available

Calculated transition radiation fields, analytical model based on reciprocity of CL detection and plane wave illumination, calculated phase rotation at singularity position, CL spectrum of a hole, Stokes mapping procedures, and signal intensity (PDF)
