# Supplementary material for: Controllable Chiral Light Generation and Vortex Field Investigation Using Plasmonic Holes Revealed by Cathodoluminescence
Source: Nano Lett. 2024 Jan 4;24(3):929–34. doi: 10.1021/acs.nanolett.3c04262 (PMC10811657; doi:10.1021/acs.nanolett.3c04262)
Supplement: Supplementary file 1 — nl3c04262_si_001.pdf [file nl3c04262_si_001.pdf]

# Supporting Information for

# Controllable chiral light generation and vortex field investigation using plasmonic holes revealed by cathodoluminescence

*Takumi Sannomiya*[1], *Taeko Matsukata*[1], *Naoki Yamamoto*[1]

AUTHOR ADDRESS

[1] Department of Materials Science and Technology, Tokyo Institute of Technology, 4259 Nagatsuta Midoriku, Yokohama 226-8503, Japan

# S1. Calculated transition radiation field

In Fig.2 in the main text, calculated transition radiation fields (TR) for a silver surface at the photon wavelength of 500 nm are shown. Here, we show TR fields in different conditions such as for a bottom carbon surface (Fig.S1a) and for a different photon wavelength (Fig.S1b). TR from carbon (Fig.S1a, left) shows a shifted phase compared to TR from silver (Fig.2d). Thus, inclusion of carbon as the surface layer would shift the TR at the bottom from the bulk silver. We also note that the propagation of the surface plasmon polariton (SPP) on the carbon surface is suppressed due to the absorptive nature of carbon.

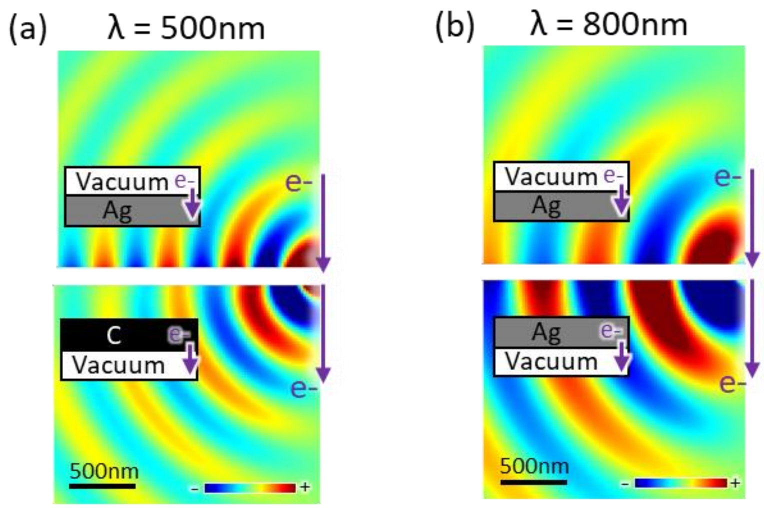


**Figure S1.** Calculated magnetic fields of transition radiation perpendicular to the screen. (a) Upper radiation from a bulk silver surface and lower radiation from a bulk carbon surface, calculated for the wavelength of 500 nm. (b) Upper and lower radiations from a bulk silver surface calculated for the wavelength of 800 nm.

## S2. Analytical model based on reciprocity of CL detection and plane wave illumination

Considering the reciprocity between cathodoluminescence (CL) detection and plane wave illumination, as described in Fig.4a in the main text and Fig.S2a below, we model the three-hole system of Fig.4 in the main text by three dipole sources representing the holes and a plane wave representing TR at each film position (Fig.2Sb). The field $E$ at position $\mathbf{r}$ on the film surface can be represented as,

$$E(\mathbf{r}) = \sum_{n=1}^{3} A_n \frac{\exp\left[\mathrm{i}\left\{\mathbf{k_{spp}} \cdot (\mathbf{r} - \mathbf{r}_n) + \mathbf{k} \cdot \mathbf{r}_n\right\}\right]}{\sqrt{r}} + A_{\mathrm{TR}} \exp(\mathrm{i}\mathbf{k} \cdot \mathbf{r}).$$

The first sum term corresponds to the three 2D dipoles located at position $\mathbf{r}_n$ and the second term to the plane wave (or transition radiation). $\mathbf{k_{spp}}$ and $\mathbf{k}$ are the wavevectors of the surface plasmon polariton and of the plane wave respectively. The coefficient $A$ is the complex amplitude of each wave source. For 2D dipoles, this coefficient includes the in-plane azimuthal angular dependence according to the polarization. To reproduce the results of Fig.4 of the main text, we adjusted the amplitudes while those of the left two holes of the same size were set to the same values.

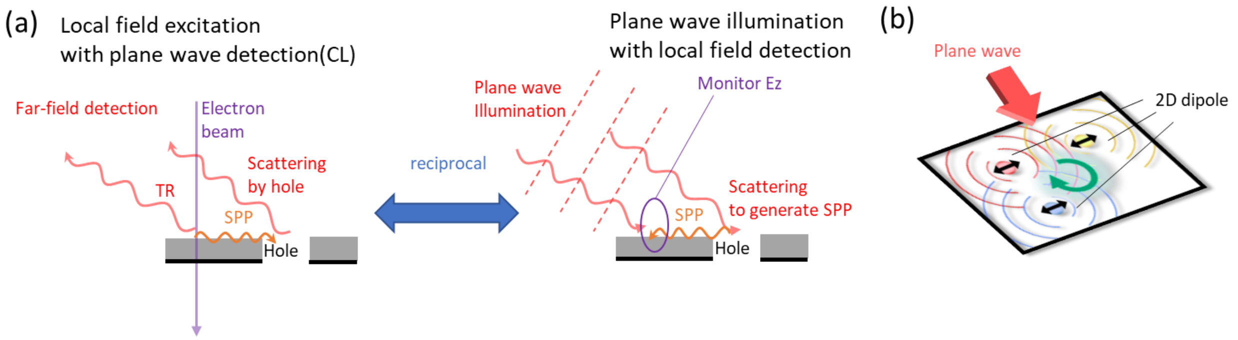


**Figure S2.** (a) Illustration of the reciprocity between far-field cathodoluminescence detection (left) and field-monitoring with plane wave illumination (right). (b) Schematics of the calculation model.

## S3. Calculated phase rotation at singularity position

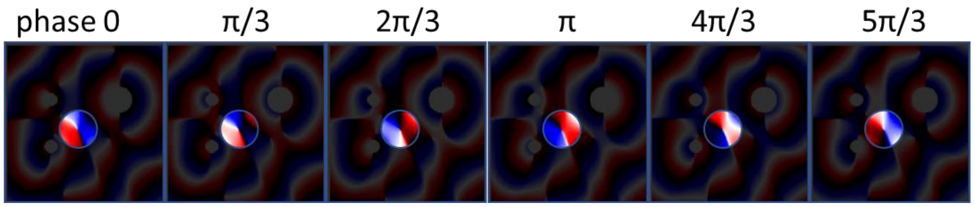


**Figure S3.** Calculated phase distribution plot of the *s*-polarized field at different off set phase values to see the phase rotation around the singularity position. To focus on this spot, other areas are shaded.

## S4. CL spectrum of a hole

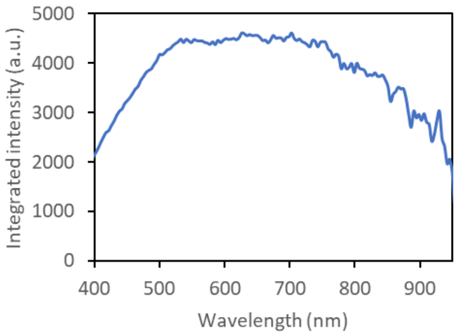


**Figure S4.** CL spectrum of an isolated 500 nm hole integrated over all the detection angles measured with *s*-polarization to eliminate the effect of TR.

## S5. Stokes mapping procedures

The Stokes parameters ($S_{0\text{-}3}$) can be obtained from the intensities of the polarized signal $I_\zeta$ (polarization condition: $\zeta$ = s(90°), p(0°), 45°, -45°, RCP, LCP ) as,

$$S_0 = I_{\text{non}} = I_{\text{s}} + I_{\text{p}},$$

$$S_1 = I_{\text{s}} - I_{\text{p}} = I_{\text{non}}\, p \cos 2\alpha \cos 2\eta,$$

$$S_2 = I_{45^\circ} - I_{-45^\circ} = I_{\text{non}} p \sin 2\alpha \cos 2\eta = 2 I_{\text{s}} I_{\text{p}} \cos\delta,$$

$$S_3 = I_{\text{RCP}} - I_{\text{LCP}} = I_{\text{non}}\, p \sin 2\eta = 2 I_{\text{s}} I_{\text{p}} \sin\delta\ ,$$

where $p = \sqrt{S_1^2 + S_2^2 + S_3^2}/S_0$. We define the ellipticity angle $\eta$ and orientation angle $\alpha$ as in Figure 1e. The ellipticity angle $\eta$ ranges from -π/4 to π/4 and is defined such that positive and negative values correspond to RCP and LCP, respectively. The phase difference $\delta$ between the p- and s-polarization fields ($I_{0^\circ}$ and $I_{90^\circ}$) can be calculated as

$$\delta = \arg(S_3/S_2).$$

We correct the position shift of the image of each polarization, which is caused by sample drifting, and normalize the intensity of the photon map of each polarization. The position of each photon map is adjusted so that the simultaneously obtained STEM images completely overlap. The intensity normalization of each image is also performed for the Stokes calculation, assuming the following two conditions:

1) The integrated intensity over all the mapping area is equal to that of the geometrically symmetric measurement with respect to the *xz*-plane, namely $\iint I_{45^\circ} ds = \iint I_{-45^\circ} ds$ and $\iint I_{RCP} ds = \iint I_{LCP} ds$.

2) The sum of the integrated intensity of orthogonal polarizations should be equal to the non-polarized intensity, i.e. $\iint I_s ds + \iint I_p ds = \iint I_{45^\circ} ds + \iint I_{-45^\circ} ds = \iint I_{RCP} ds + \iint I_{LCP} ds$.

These conditions originate from the symmetry of the measurement system with respect to the *xz*-plane. The above formulations deduce simpler normalization relations of the integrated intensities for ±45° and circular polarizations as $\iint I_{45^\circ} ds = \iint I_{-45^\circ} ds = \iint I_{RCP} ds = \iint I_{LCP} ds$.

## S6. Signal intensity

Figure S5a shows the emission probability of TR and SPP on a silver surface calculated for 80 keV electron according to the literature.[1] With the electron beam current of 1 nA, as used in this study, the generated photons by TR are ~ 2 × 106 /s for the shown wavelength range (400-750nm). The rate of SPPs is calculated as ~ 6 × 106 /s in the same wavelength range. The number of the detected photons are reduced by selecting the wavelength range and the detection solid angle. As SPPs propagate from the electron beam position toward the hole in a circular

manner, the SPP intensity is reduced by the distance as well as by the material loss, as shown in Fig.S5b. For the longer wavelength range (>500 nm), the propagation length is large enough, indicating that the material loss is not significant for this measurement. In contrast, the loss effect is more clearly visible for the plots of 400 nm wavelength in Figs. 2 and 3 in the main text.

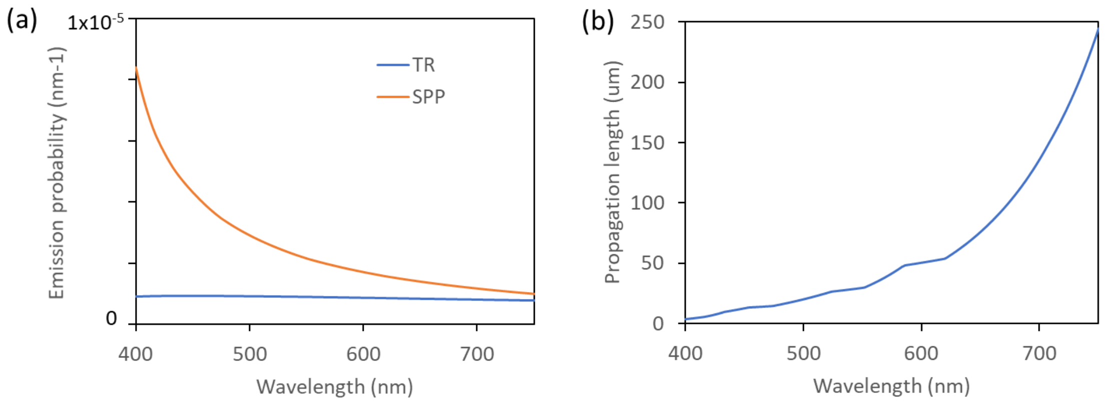


**Figure S5.** (a) Emission probability of TR and SPP on a silver surface calculated for 80 keV electron incidence. (b) Propagation length of the SPP on silver. Literature values for the material parameters are used.[2]

**References**


[1] F.J. García  de Abajo, Optical excitations in electron microscopy, Reviews of Modern Physics 82(1) (2010) 209-275.

[2] P.B. Johnson, R.W. Christy, Optical Constants of Noble Metals, Physical Review B 6(12) (1972) 4370-4379.
